# Dietary 25-Hydroxycholecalciferol Supplementation as a Vitamin D_3_ Substitute Improves Performance, Egg Quality, Blood Indexes, Jejunal Morphology, and Tibia Quality in Late-Phase Laying Hens

**DOI:** 10.3390/ani14060878

**Published:** 2024-03-13

**Authors:** Shan Gao, Kai Qiu, Junjie Zheng, Haijun Zhang, Jing Wang, Xiaolong Qi, Shugeng Wu

**Affiliations:** 1 Laboratory of Quality and Safety Risk Assessment for Animal Products on Feed Hazards (Beijing), the Ministry of Agriculture and Rural Affairs, Institute of Feed Research, Chinese Academy of Agricultural Sciences, Beijing 100081, China; caasgs20141114@126.com (S.G.); qiukai@caas.cn (K.Q.); zhanghaijun@caas.cn (H.Z.); wangjing@caas.cn (J.W.); 2 Beijing Agricultural Products Quality and Safety Center, the Ministry of Agriculture and Rural Affairs, Beijing 100020, China; 13521296211@163.com; 3 Animal Science and Technology College, Beijing University of Agriculture, Beijing 102206, China

**Keywords:** 25-hydroxycholecalciferol, calcium absorption, performance, tibia health, late-phase laying hens

## Abstract

**Simple Summary:**

Improving the production performance and egg quality of laying hens in the late-laying period has become an urgent problem. This study confirmed that dietary supplementation of 25-hydroxycholecalciferol (25-OHD_3_) instead of vitamin D_3_ (VD_3_) improved production performance and egg quality, maintained the jejunal morphology, and enhanced tibia quality by increasing the concentration of circulating 25-OHD_3_ and Ca absorption in late-laying-period hens. The superiority of 25-OHD_3_ over VD_3_ indicates that it has the potential to prevent an age-induced decrease in production performance and osteoporosis in laying hens, thereby contributing to extending the feeding cycle of laying hens.

**Abstract:**

This study aimed to investigate whether a dietary 25-OHD_3_ addition improved the performance, egg quality, blood indexes, antioxidant status, jejunal morphology, and tibia quality of aged laying hens compared to a dietary VD_3_ addition. A total of 270 Hy-Line Brown laying hens at 55 wk of age were randomly assigned to three dietary treatments with six replicates (15 birds per replicate with 3 birds per cage). Chickens were fed a corn–soybean meal diet supplementation of 4000 IU/kg VD_3_ (control group), 50 μg/kg 25-OHD_3_ and 2000 IU/kg VD_3_ (experimental group 1), or 50 μg/kg 25-OHD_3_ and 4000 IU/kg VD_3_ (experimental group 2) for 12 weeks. The results demonstrated that 25-OHD_3_ caused a significant increase in the laying rate, especially in the 50 μg/kg 25-OHD_3_ + 2000 IU/kg VD_3_ group; the laying rate reached the maximum compared with other groups after 12 weeks (*p* < 0.05). However, there were no significant effects on the average egg weight, average daily feed intake, or feed-to-egg ratio (*p* > 0.05). A dietary supplementation of 50 μg/kg 25-OHD_3_ and 2000 IU/kg VD_3_ provided an improved eggshell strength, thick albumen height, and Haugh unit after 12 weeks (*p* < 0.05). Further analysis of the blood indexes showed that alanine aminotransferase, aspartate aminotransferase, alkaline phosphatase, calcium, and phosphorus were enhanced significantly in the 50 μg/kg 25-OHD_3_ + 2000 IU/kg VD_3_ group, while the content of total bilirubin decreased significantly (*p* < 0.05). In addition, the 25-OHD_3_ addition in diets improved the calcium and phosphorus contents in the serum (*p* < 0.05). The concentrations of 25-OHD_3_, parathyroid hormones, follicle-stimulating hormone, and progesterone were increased in the 50 μg/kg 25-OHD_3_ + 2000 IU/kg VD_3_ group, and the levels of cortisol, calcitonin, bone gla protein, and endotoxin in the serum reached a minimum in the 50 μg/kg 25-OHD_3_ + 4000 IU/kg VD_3_ group (*p* < 0.05), which constitutes an advantage for the aged laying hens. The antioxidant enzyme activities and free radical scavenging abilities in the 50 μg/kg 25-OHD_3_ + 2000 IU/kg VD_3_ group increased markedly, and the MDA level decreased significantly in the 50 μg/kg 25-OHD_3_ + 4000 IU/kg VD_3_ group (*p* < 0.05). Improvements in jejunal morphology and intestinal integrity resulted in an increased villi-length-to-crypt-depth ratio in the 50 μg/kg 25-OHD_3_ + 2000 IU/kg VD_3_ group (*p* < 0.05). Dietary 50 μg/kg 25-OHD_3_ and 2000 IU/kg VD_3_ additions improved the tibia quality, including fresh tibia weight, strength, mineral content (Ca), and trabeculae area (*p* < 0.05). Taken together, compared with the dietary VD_3_ addition, dietary supplementation of 25-OHD_3_ supported a stable physiological status for sustained egg production, egg quality, and bone quality in late-phase laying hens, and the addition levels of 50 μg/kg 25-OHD_3_ and 2000 IU/kg VD_3_ had the best effect. Therefore, this could provide a theoretical basis for the use of 25-OHD_3_ as a substitute forVD_3_.

## 1. Introduction

The laying period from 45 weeks of age to 72 weeks of age is often referred to as the late phase of hen laying. This cycle of production is often linked with increased reactive oxygen species (ROS) production, declined production performance, reduced antioxidant capacity, low secretion of reproductive hormones, and impaired intestinal oxidation status, which culminate in poor eggshell quality/irregular shell formation [1,2]. Laying hens absorb calcium from the intestine or via bone resorption. About 30–40% of the calcium released from bones is deposited into the eggshells of birds in the late-laying phase, which is reflected in a lower bone mineral content and density [3,4]. Laying hens in the peak laying period, reared on a low-Ca (1.5%) diet, had higher rates of broken eggs and lower body weights, feed consumption, and laying rates compared to birds fed normal-Ca (3.9%) diets [5]. In the late-production phase, increasing dietary Ca from 3.5% to 4.5% linearly improved the laying rate of birds [6]. Mineral reserves are critical for eggshell quality and bone integrity in older laying hens since intestinal Ca absorption diminishes with age [7]. Consequently, Ca supplementation is required throughout the production cycle of laying hens.

Moreover, the skeletal health of laying hens has gained research focus. According to a previous study, 30% of laying hens suffer bone fractures before slaughter, reducing their economic value [8]. Currently, the cage housing system is the most popular raising strategy for layers in China due to its cost advantages, i.e., because there are no significant effects on egg deposition patterns and egg quality, except for improving the state of the feathers of laying hens compared with some welfare cages [9]. However, the cage leg problem may be a challenge for old laying hens because the late phase of production is linked with incidences of osteoporosis [10]. Also, caged hens with high laying rates develop “cage layer fatigue” (CLF), which often leads to structural bone loss and fractures in various bone areas at the end of the laying cycle [11]. Higher laying rates and low-Ca diets may cause loss of parenchymal bone and osteoporosis, the main bone disease of laying hens [11,12].

Optimal performance in aged laying hens requires nutritional interventions that can maintain antioxidant status, Ca requirements, and homeostasis, and reduce negative impacts on performance, egg quality, and bone health. Vitamin D_3_ (VD_3_), also known as cholecalciferol, promotes the intestinal absorption of Ca and regulates mineral and skeletal homeostasis [13,14]. Previously, supplementing laying hens with VD_3_ (from 1681 IU/kg to 35,014 IU/kg) from 0 to 68 weeks increased the laying rate, egg quality attributes (Haugh unit, eggshell breaking strength), and bone quality linearly, but these positive effects were reversed at 68,348 IU/kg [15]. The inclusion of an appropriate amount of VD_3_ in the diet can boost the laying rate and tibial breaking strength [16]. These findings suggest that dietary VD_3_ supplementation increased the laying performance within a certain inclusion range. However, dietary supplementation of higher levels VD_3_ may have a detrimental effect on the performance of laying hens.

VD_3_ is activated and converted into an active metabolite (25-OHD_3_) in the liver [17], but the conversion may be impaired in old laying hens. 25-OHD_3_ had higher biological activity than VD_3_, less side effects, and greater stability than 1,25-dihydroxycholecalciferol, and dietary 25-OHD is characterized by a better strong affinity and absorption efficiency with Vitamin D binding protein [18]. Nowadays, 25-OHD_3_ has been validated as a source of VD_3_ and is widely utilized in poultry production. Previous studies have shown that the addition of 25-OHD_3_ in the diet has positive effects on the production performance and bone health of laying hens. A study showed that long-term (from 0–95 weeks) replacement of VD_3_ with 25-OHD_3_ (equivalent from VD_3_) significantly increased the circulating 25-OHD_3_ concentration, improved bone health, and provided more space for mineral deposition during the late-production phase of laying hens [19]. However, another study showed that compared with 62.5 μg/kg doses of VD_3_, supplementary 125 μg/kg doses of VD_3_ or 25-OHD_3_ improved the performance and egg quality, while there were no remarkable differences among the two groups [20].

Although 25-OHD_3_ is a bioactive dietary form (1 μg of cholecalciferol is equivalent to 40 IU of VD_3_), the effects of dietary 25-OHD_3_ supplementation as a vitamin D_3_ substitute on late-laying hens are unknown. Given the aforementioned findings, this study aimed to verify the effects of dietary 25-OHD_3_ as a VD_3_ alternative on performance, egg quality, blood indices, jejunal morphology, and tibia quality. To further explore whether dietary supplementation of 25-OHD_3_ can be used as a nutritional strategy to improve bone health in aging progress by promoting the absorption and deposition of calcium and phosphorus.

## 2. Materials and Methods

The experimental protocol used in this study was approved by the Animal Care and Use Committee (IFR-CAAS-20220401) of the Institute of Feed Research of the Chinese Academy of Agriculture Science.

### 2.1. Birds and Housing

During the feeding trial, laying hens were randomly housed in 3-tier battery cages (40 cm × 40 cm × 35 cm) and exposed to 16 h of incandescent light and 8 h of darkness on a daily basis, and the light intensity was 20 lux. The birds were fed twice daily (8:00 and 14:00) and allowed ad libitum access to water and treatment diets during the experimental period. The average temperature in the laying hen house was 21 ± 3 °C with natural ventilation and the room’s relative humidity at 50–80%. The birds were in good health throughout the feeding trial.

### 2.2. Experimental Design and Diets

A total of 270 Hy-Line Brown laying hens (55 weeks old, 2.08 ± 0.09 kg body weight) were randomly assigned to three treatment groups with six replicates per treatment (15 laying hens per replicate). The control group was fed a basal diet supplemented with 4000 IU/kg VD_3_. Experimental group 1 was fed a basal diet with a supplementation of 50 μg/kg 25-OHD_3_ and 2000 IU/kg VD_3_. Experimental group 2 was fed a basal diet supplemented with 50 μg/kg 25-OHD_3_ and 4000 IU/kg VD_3_. The product with 0.05% of 25-OHD_3_ was purchased from Zhejiang Jin Langbo Pharmaceutical Co., Ltd. (Xinchang, China) and prepared by using the biotransformation method. The composition and nutrient levels of the corn–soybean meal-based diets are shown in Table 1. All hens were fed a basal diet for 1 week and then assigned to dietary treatments for 12 weeks.

### 2.3. Sample Collection and Measurements

At weeks 4, 8, and 12 of the trial, five eggs were randomly selected from each replicate; thus, a total of 270 eggs were used for the egg quality determination during the three assessment periods, and egg quality detection was completed within 3 h after egg collection (the storage temperature was 24–26 °C). At the end of the experiment period, 6 birds per treatment (1 bird per replicate) were randomly selected and fasted for 8 h prior to slaughter. Blood samples were collected from the wing vein of each bird, and the serum was separated by centrifugation at 3000× *g* for 10 min and stored at −20 °C for biochemical parameter analysis. The selected birds were sacrificed by cervical dislocation, and the jejunum samples were collected and fixed in a 4% paraformaldehyde solution for 48 h. Also, the left and right tibias of each bird were collected and cleaned of all tissue (muscle, tendon, and fat). The left tibia was fixed with 4% paraformaldehyde and decalcified with 10% EDTA solution. The right fresh tibia samples were weighed with an electronic scale (accuracy of 0.01 g) after removing the fat and tissue.

### 2.4. Production Performance and Egg Quality Analysis

Production performances (egg number, total egg weight, feed intake, and unqualified eggs (egg weight < 50 g or 75 g)) were recorded daily for each replicate, accordingly. The feed-to-egg ratio was calculated as the ratio of feed intake (grams) to the total egg weight (grams). The laying rate was calculated as the total number of eggs/total number of hens × 100%. The average egg weight, average daily feed intake, laying rate, and feed-to-egg ratio were calculated for wks 1–4, wks 5–8, and wks 9–12. The average egg weight was calculated as the total egg weight/total number of eggs. The egg weight, egg yolk weight, and albumen weight were measured by an electronic scale (accuracy 0.01). The ratio of egg yolk or albumen was calculated as their weight/egg weight × 100%. The length and width of eggs were measured using an electronic vernier caliper (Shanghai Shenhan Measuring Tools Co., Ltd., Shanghai, China), and the egg shape index was calculated using the length/width ratio. Also, the eggshell strength was determined with an eggfore reader (ORKA Food Technology Ltd., Ramat HaSharon, Israel). In addition, the Haugh unit and thick albumen height were determined with an automated egg quality analyzer (ORKA Food Technology Ltd., Ramat HaSharon, Israel).

### 2.5. Blood and Serum Biochemical Parameters Analyses

The blood biochemical indices include the activities of alanine aminotransferase (ALT), aspartate aminotransferase (AST), and alkaline phosphatase (ALP). Moreover, the levels of albumin (ALB), uric acid (UA), creatinine (CRE), glucose (GLU), total bilirubin (T-BIL), calcium (Ca), and phosphorus (P) were determined by an ultraviolet spectrophotometer. These assays were performed using a commercial kit (Shanghai Kehua Bio-Engineering Co., Ltd., Shanghai, China), and all protocols were followed in accordance with the manufacturer’s instructions.

The activities of glutathione peroxidase (GSH-Px), superoxide dismutase (SOD), catalase (CAT), the abilities of scavenging radicals, the total antioxidant capacity (T-AOC), and the product of lipid metabolism (MDA) concentration were analyzed using the commercial kits (Nanjing Jiancheng Bio-Engineering Institute, Nanjing, China) by a microplate reader. In addition, the hormone levels included 25-OHD_3_, follicle-stimulating hormone (FSH), testosterone (T), progesterone, parathyroid hormone (PTH), bone gla protein (BGP), calcitonin (CT), carbonic anhydrase (CA), cortisol (COR), diamine oxidase (DAO), and endotoxin (ET). These assays were performed using the chicken ELISA kits (Nanjing Jiancheng Bio-Engineering Institute, Nanjing, China) according to the instructions.

### 2.6. Intestinal Morphology Analysis

Intestinal morphology analysis was conducted using a previously described method [21]. Briefly, jejunum tissues were dehydrated, embedded in paraffin, and sectioned into 4 μm thick sections for histological examination. After HE (hematoxylin–eosin) staining, each stained section was photographed under a 40× magnification with a digital microscope (Olympus Corporation, Tokyo, Japan), and three typical fields of view were selected for each stained section. The villus height and crypt depth were measured using the medical image analysis software ImagePro-Plus 7.0 (Media Cybernetics lnc., Rockville, MD, USA). Finally, the ratio of villi-length-to-crypt-depth (V/C) was calculated.

### 2.7. Tibial Quality Analysis

The left tibia samples were fixed with 4% paraformaldehyde and decalcified with 10% EDTA solution, then dehydrated and embedded in paraffin. The paraffin blocks were sectioned into 5 μm thick sections, stained with toluidine blue, and a histological observation was performed under the scan at 200× magnification. The number of bone trabeculae and area of bone trabeculae were evaluated by the medical image analysis software ImagePro-Plus 7.0 (Media Cybernetics lnc., Rockville, MD, USA).

Furthermore, the tibia strength was measured with a texture analyzer (Food Technology Corporation, Sterling, VA, USA). A three-point bending test of the metaphyseal tibia was done with a supporting distance of 30 mm and a test speed of 10 mm/min [22]. According to previous study methods, the ash, calcium (Ca), and phosphorus (P) contents of tibia were determined [23]. Briefly, the bone samples were placed in an oven at 105 °C for 48 h to eliminate moisture and then weighed. Secondly, after defatting with petroleum ether (Sinopharm Chemical Reagent Co., Ltd., Beijing, China), the dried bone was carbonized at 600 °C for 3 h. Next, after ashing at 550 °C for 12 h in a muffle furnace, samples were ground into powder, and the percentage of ash was determined.

For further analysis, a tibial powder sample (0.2 g) was added to the conical flask, 5 mL of nitric acid (Sinopharm Chemical Reagent Co., Ltd., Shanghai, China) was fully mixed with the sample, and it was digested at 220 °C in an electric sand bath for 3 h until there was no turbidity in the solution. After cooling at room temperature, the samples were diluted 625 times with ultrapure water and filtered through a 0.22 μm filter membrane. The contents of Ca and P in the samples were detected by an inductively coupled plasma atomic emission spectrometer (Agilent Technologies lnc., Palo Alto, CA, USA).

### 2.8. Statistical Analysis

Statistical analysis was performed using SPSS 20.0 (IBM Corp., Armonk, NY, USA). The data relating to the effect of dietary 25-OHD_3_ levels on performance, egg quality, blood indexes, intestinal morphology, and tibia quality in the late-laying period were analyzed by one-way ANOVA, the means were compared using Tukey’s multiple test, and significant differences were considered at *p* < 0.05.

## 3. Results

### 3.1. Production Performance

Production performance is one of the important indicators for evaluating poultry production efficiency. The effects of dietary supplementation of 25-OHD_3_ on the production performance of laying hens are listed in Table 2. The results showed that dietary supplementation of 25-OHD_3_ significantly increased the laying rate (*p* < 0.05) compared to the control group. Particularly, the dietary addition of 50 μg/kg 25-OHD_3_ and 2000 IU/kg VD_3_ significantly increased the laying rate at weeks 8 and 12 compared to other groups (*p* < 0.05). However, there were no significant effects of the dietary 25-OHD_3_ addition on the average egg weight, average daily feed intake, and feed-to-egg ratio related to the control group throughout the feeding trial (*p* > 0.05). In addition, no mortality occurred throughout the test period.

### 3.2. Egg Quality Assessment

In this study, the effects of dietary 25-OHD_3_ supplementation on the egg quality of laying hens are listed in Table 3. The enhancement effects of the dietary 50 μg/kg 25-OHD_3_ and 4000 IU/kg VD_3_ addition on eggshell strength was obvious at the end of week 8, and eggshell strength improved significantly in the 50 μg/kg 25-OHD_3_ + 2000 IU/kg VD_3_ group at week 12 (*p* < 0.05). We further found that dietary supplementation of 25-OHD_3_ caused a significant increase in the thick albumen height and Haugh unit (*p* < 0.05), and the value reached the maximum in the 50 μg/kg 25-OHD_3_ + 2000 IU/kg VD_3_ groups at week 12. Notably, there were no significant effects on the eggshell strength, thick albumen height, and Haugh unit in the 50 μg/kg 25-OHD_3_ + 2000 IU/kg VD_3_ group or 50 μg/kg 25-OHD_3_ + 4000 IU/kg VD_3_ group. Moreover, there were no significant effects of dietary treatments on the egg yolk weight, albumen weight, egg yolk rate, albumen rate, and egg shape index (*p* > 0.05).

### 3.3. Serum Biochemistry Analysis

The effects of dietary 25-OHD_3_ supplementation on the serum biochemical indices are presented in Table 4. There were notable effects of the dietary addition of 50 μg/kg 25-OHD_3_ and 2000 IU/kg VD_3_ on the ALT activity, ALB level, and P level (*p* < 0.05). Moreover, Ca levels in the 50 μg/kg 25-OHD_3_ + 4000 IU/kg VD_3_ groups enhanced significantly (*p* < 0.05). However, the T-BIL level in the 50 μg/kg 25-OHD_3_ + 2000 IU/kg VD_3_ group and the ALP level in the 50 μg/kg 25-OHD_3_ + 4000 IU/kg VD_3_ group declined remarkably, respectively (*p* < 0.05). Moreover, no remarkable differences in the activity of AST were observed among the dietary groups (*p* > 0.05). Moreover, compared to the control group, the levels of UA, CRE, and GLU in serum remained similar across the dietary treatments (*p* > 0.05).

### 3.4. Serum Hormone Concentration

The effects of dietary 25-OHD_3_ supplementation on the concentration of serum hormones are shown in Table 5. The concentration of 25-OHD_3_ in the serum was significantly increased in the 50 μg/kg 25-OHD_3_ + 2000 IU/kg VD_3_ group (*p* < 0.05). In addition, a similar trend for enhancement effects in the 50 μg/kg 25-OHD_3_ + 2000 IU/kg VD_3_ bird-fed group was notable for concentrations of hormones, including FSH, progesterone, T, and PTH (*p* < 0.05). Nevertheless, it is noteworthy to mention that the dietary inclusion of 25-OHD_3_ remarkably decreased concentrations of -COR, BGP, CT, and ET (*p* < 0.05) compared to the control group. There were no significant effects in the concentrations of CA and DAO among all the groups (*p* > 0.05).

### 3.5. Antioxidant Capacity

The effects of dietary 25-OHD_3_ supplementation on the serum antioxidant parameters are presented in Table 6. The activities of GSH-Px, CAT, and SOD in the 50 μg/kg 25-OHD_3_ + 2000 IU/kg VD_3_ groups were higher than that in the control group, while the MDA level was drastically reduced in the 50 μg/kg 25-OHD_3_ + 4000 IU/kg VD_3_ group (*p* < 0.05). Moreover, the abilities of scavenging superoxide anions and scavenging hydroxyl radicals were enhanced markedly (*p* < 0.05) in the treatment groups compared to the control group. However, there were no significant effects on the T-AOC levels among the groups (*p* > 0.05). Thus, dietary supplementation of 50 μg/kg 25-OHD_3_ and 2000 IU/kg VD_3_ provided the highest antioxidant capacity.

### 3.6. Intestinal Morphology

**Figure 1:**
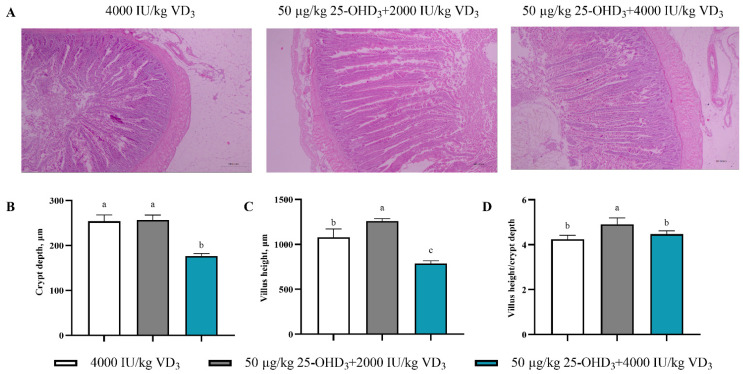
Effects of dietary supplementation of 25-OHD_3_ on intestinal morphology of laying hens. (**A**) The jejunum was stained with HE solution (300 microns, ×40). (**B**) Crypt depth. (**C**) Villus height. (**D**) Villi-length-to-crypt-depth ratio. The different superscript small letters were judged as a significant difference (*p* < 0.05).

The morphology of the jejunum in laying hens is shown in Figure 1. The dietary addition of 25-OHD_3_ presented an intact intestinal structure with longer villi and regular crypt morphology (*p* < 0.05) compared to the control group. Additionally, the under 50 μg/kg 25-OHD_3_ + 2000 IU/kg VD_3_ bird groups, in comparison to the control group, had a significant reduction in crypt depth and notable increases in the villi-length-to-crypt-depth ratio (*p* < 0.05).

### 3.7. Tibia Quality

**Figure 2:**
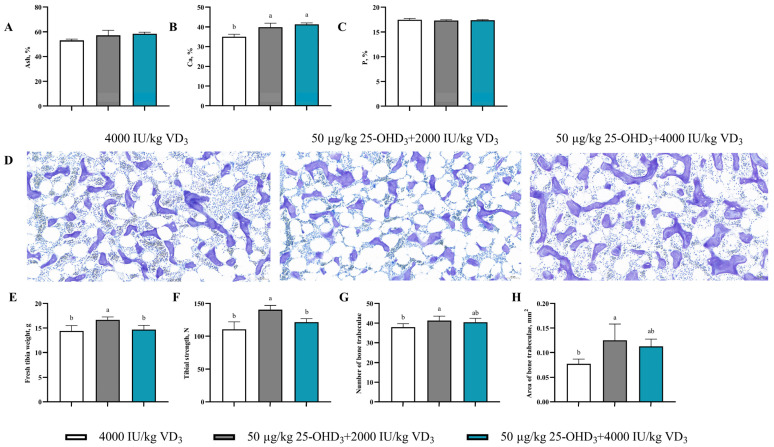
Effects of dietary supplementation of 25-OHD_3_ on tibia quality of laying hens. The content of (**A**) ash, (**B**) Ca, and (**C**) P in the tibia. (**D**) Effect of dietary 25-OHD_3_ on tibia histopathology of laying hens: the medullary trabecular bone was stained with toluidine blue solution (×200). (**E**) Fresh tibia weight. (**F**) Tibia strength. (**G**) The number of trabecular. (**H**) The area of trabeculae bone. The different superscript small letters were judged as a significant difference (*p* < 0.05).

As shown in Figure 2, the Ca content in the 50 μg/kg 25-OHD_3_ + 2000 IU/kg VD_3_ group or 50 μg/kg 25-OHD_3_ + 4000 IU/kg VD_3_ groups were remarkably increased (*p* < 0.05) compared to the control group, but there were no significant variations for the content of ash and P (*p* > 0.05) in the tibia samples. Moreover, a histopathology evaluation of the tibia revealed the presence of a cortical bone structure and trabeculae in the tibia as closely arranged. The number and area of trabeculae in the 50 μg/kg 25-OHD_3_ + 2000 IU/kg VD_3_ group were significantly increased relative to the control group (*p* < 0.05). In addition, dietary 50 μg/kg 25-OHD_3_ and 2000 IU/kg VD_3_ supplementation increased the fresh tibia weight and strength (*p* < 0.05).

## 4. Discussion

Achieving optimal laying rates, eggshell quality, and bone health are major issues of concern for the poultry industry with respect to laying hens in the late phase of the production cycle. Previous reports showed that the laying rate and egg quality (including the eggshell strength and thickness) decline with the age increase of hens [24]. Consequently, about 10–15% of produced eggs are lost due to poor eggshell quality [25]. Laying hens with high egg production rates may develop avian osteoporosis due to the resorption of both medullary and structural bone for eggshell development during the late phase of the cycle [11]. Interestingly, nutritional strategies can mitigate the adverse impacts of age on production performance, bone health, and egg quality during the late-laying phase. The current study provides support for the dietary addition of 25-OHD_3_ as a VD_3_ substitute to enhance performance, egg quality, physiology, and bone health in birds at the late-laying phase.

In the current study, dietary supplementation of 25-OHD_3_ significantly increased the laying rate compared to the VD_3_-supplemented group. The previous study reported that dietary supplementation of an equal amount of 25-OHD_3_ had similar effects to VD_3_’s significantly increased laying rate and decreased unqualified egg rate [20]. In another study, dietary supplementation of 25-OHD_3_ increased the production rate and feed conversion ratio compared with a dietary VD_3_ addition in aged laying hens [13]. Thus, the dietary addition of 25-OHD_3_ or 25-OHD_3_ and VD_3_ combination exerted a superior positive effect on the laying rate of late-laying phase hens compared to the normal VD_3_ group, which is due to more demand for more active 25-OHD_3_ to maintain the production performance in this laying phase. In another study, dietary 25-OHD_3_ did not influence the laying rate, egg weight, and feed efficiency of laying hens [26,27]. Long-term dietary supplementation of 25-OHD_3_ at 69 or 125 µg/kg as a substitute to VD_3_ had no significant effects on the laying rate, egg weight, and feed efficiency but enhanced the feed intake [28]. The variations observed could be due to the supplement dosage. Undoubtedly, sustained egg production at more than 60 weeks of age, suggests that future research directions should focus on the dynamics of calcium requirements for laying hens throughout the egg production cycle and the underlying physiology that supports better laying rates and egg quality.

Egg quality traits, including the eggshell strength and albumen quality, are key indicators for consumers’ perceptions and demand for table eggs. Further analysis in the study showed that a dietary 50 μg/kg 25-OHD_3_ and 2000 IU/kg VD_3_ addition caused a remarkable increase in eggshell strength and thick albumen height. This corroborates the previous findings that various sources of vitamin D, VD_3_, 25-OHD_3_, and 1,25(OH)_2_D_3_ caused a significant increase in the Haugh unit of Hy-Line W-36 laying hens at 80 weeks of age [13]. In addition, partial or complete replacement of VD_3_ with 25-OHD_3_ in diets enhanced the eggshell quality and reduced the rate of broken eggs [29], whereas the inclusion of 25-OHD_3_ in a basal diet containing VD_3_ improved the eggshell thickness [28]. Eggshell brightness and eggshell weight were improved with dietary 25-OHD_3_ [27]. Another study reported that a dietary 25-OHD_3_ addition improved the eggshell thickness with a high stocking density of laying hens. Supplementation of 25-OHD as a substitute to VD_3_ in the diets of laying hens (from 1–70 weeks) enhanced the eggshell thickness at 60 weeks of age but had no effect on other egg quality traits [28]. Another study showed that the dietary addition of different isoforms of vitamin D had no effect on the egg quality of Lohmann laying hens [30]. These results suggest that dietary supplementation with 25-OHD_3_ has positive effects on the Haugh unit, eggshell strength, and eggshell thickness compared with a VD_3_ addition. However, varying results across the research studies could be attributed to the age of laying hens, dosage of the supplement, and viability period of the functional ingredients.

Blood biochemical indices are used as indicators to evaluate normal body functions and physiology. In the current study, the dietary 25-OHD_3_ addition exerted significant effects on the activities and levels of biochemical markers, such as ALT, ALB, and ALP, while reducing the level of T-BIL. ALP is an enzyme that is primarily involved in the deposition of Ca and P and is strongly linked with the rate at which skeletal mineralization occurs in birds [31]. The reduced content of ALP could be accrued to circulating levels of 25-OHD_3_, which aid Ca metabolism and utilization, preventing bone disorders that may increase its activity. The increased content of ALB could explain the efficient transport of the minerals and hormones observable in this study, which accounts for the positive effects on the physiology of the laying hens. In our study, Ca levels in serum in the 50 μg/kg 25-OHD_3_ + 2000 IU/kg VD_3_ group and serum P levels in the 50 μg/kg 25-OHD_3_ + 4000 IU/kg VD_3_ group enhanced significantly. This corroborates with a previous report that the dietary addition of different isoforms of VD_3_ increased the utilization of Ca and P in laying hens [30]. Also, a study showed that a dietary 69 μg/kg 25-OHD_3_ addition increased the content of Ca in serum but had no effect on the P content [32]. However, a long-term deficiency or low content of VD_3_ in the diet led to a drastic decline in the Ca and P contents in the serum of laying hens, and these variations were notable for various genotypes [33]. The contents of Ca and P in serum reflect the nutritional status of these minerals in the body. Therefore, 25-OHD_3_ improves Ca and P retention and absorption, which will facilitate the absorption and utilization of these minerals compared with VD_3_.

Also, the homeostasis of Ca content in the body is achieved through intestinal absorption, renal excretion, Ca deposition into the eggshell, and bone metabolism, which are jointly regulated by PTH, CT, VD_3_, and sex hormones [34]. The critical value of the Ca level in enhancing reproductive function in aged laying hens was reported [35]. In the current study, the addition of 50 μg/kg 25-OHD_3_ and 2000 IU/kg VD_3_ in the diet caused an increase in sex hormones (FSH and progesterone), PTH level, and 25-OHD_3_ level, while levels of CORT, ET, BGP, and CT in the 50 μg/kg 25-OHD_3_ + 4000 IU/kg VD_3_ groups reduced significantly. Similar results previously reported that a dietary addition of 69 μg/kg 25-OHD_3_ reduced COR, the lipopolysaccharide level in the serum of laying hens [27]. Nevertheless, a dietary 25-OHD_3_ addition had no effect on the serum level of PTH in broiler birds [32]. Additionally, one of the key reliable indicators of vitamin D status in the body of an animal is the measurement level of 25-OHD_3_ in the circulating blood. Preceding studies showed that dietary 25-OHD_3_ supplementation enhanced the 25-OHD_3_ circulation level in the blood and promoted bone structure development for mineral deposition and bone quality during the laying period [19,27,32]. In another study, increased levels of 25-OHD_3_ in the serum of laying hens due to dietary treatments were notable for various breeds of chickens [33]. The positive effects of high circulating 25-OHD_3_ are accrued to its capacity to supply a sufficient amount of 25-OHD_3_ compared to regular vitamin D supplementation. The aforementioned findings are suggestive of adopting 25-OHD_3_ as a substitute for VD_3_ in the diet of laying hens during the late-production cycle.

Moreover, the antioxidant capacity and liver function of laying hens declined significantly during the late-production phase. In one study, increased AST activity, elevated MDA, a lipid peroxidation product, and decreased activities of CAT, GSH, GSH-Px, and SOD were observed in aged laying hens [36]. These effects imply reduced antioxidant status and tissue/organ damage due to the higher activity of AST. Higher activities of antioxidant enzymes, including SOD, CAT, and GSH-Px, inactivate the ROS; the reason may be that antioxidant enzymes have unique ways to remove excessive produced free radicals [37,38]. These effects are linked with enhancing a balance between oxidant and antioxidant systems for stable physiology and animal health. Our findings indicated the potency of dietary 50 μg/kg 25-OHD_3_ and 2000 IU/kg VD_3_ supplementation on increased antioxidant capacity and scavenging ability of free radicals compared to the control group. This is consistent with a previous study that demonstrated the antioxidant effect of dietary 25-OHD_3_. In one study with broiler birds fed diets with low Ca and P levels, dietary 25-OHD_3_ prevented oxidative stress-induced osteoporosis [32]. Also, oxidative stress (induced by restricted feeding [39]), high stocking density [40], and exposure to lipopolysaccharide challenge in broilers and laying hens, respectively, were alleviated with dietary 25-OHD_3_ [14]. In addition, 25-OHD_3_ increased the activities of antioxidant enzymes in both serum and tissue (jejunum), maintained intestinal barrier function, and caused a reduction in the levels of proinflammatory cytokines in laying hens and broiler birds [32,40]. Thus, a dietary 25-OHD_3_ addition improves the antioxidant function in birds via the upregulation of antioxidant enzymes and inhibition of oxidants.

A relationship exists between antioxidant status and bone health. Caged laying hens are often predisposed to osteoporosis and other bone problems due to oxidative stress [41], and a strong correlation between high levels of oxidative stress and osteoporosis has been reported [42]. Due to an imbalance in the oxidant–antioxidant system, reactive oxygen species can cause osteocyte death and disturb bone formation. The resultant Ca deficiency could lead to a decrease in the activity of antioxidant enzymes such as SOD [43]. Therefore, it is plausible that dietary supplementation of 25-OHD_3_ can augment mineralization and bone quality via an improved antioxidant defense system.

Furthermore, the small intestine aids Ca absorption; the transcellular and paracellular Ca transport channels are most active in the duodenum and jejunum, especially in birds [44]. Earlier studies demonstrated that healthy intestinal conditions could promote the absorption of nutrients because there were larger absorption areas and a higher turnover rate of epithelial cells [45]. Nevertheless, the functional role of intestinal morphology often declines with the age of laying hens, but nutritional interventions can modulate these changes. Our present findings show that the dietary supplementation of 50 μg/kg 25-OHD_3_ and 2000 IU/kg VD_3_ increased villi height and the villi-height-to-crypt-depth ratio compared to the VD_3_ group. This is in agreement with previous reports, which showed that dietary 25-OHD_3_ improved the expression of intestinal barrier-related genes, thus improving intestinal integrity [40]. In one study with aged laying hens, combined treatment of biscuit meal and a by-product of modified olive oil maintained intestinal morphology, improved digestive function, and increased egg production, thus counteracting the adverse effects of age on intestine morphology [46]. Therefore, we deduce that compared with VD_3_, 25-OHD_3_ may improve performance, egg quality, circulation of hormones, and mineral contents via an enhancement effect on intestinal morphology for better nutrient utilization.

A further evaluation of bone quality showed that a dietary 25-OHD_3_ addition enhanced the arrangement of bone trabeculae tightly, the number and area of trabeculae, and tibia strength. The findings are suggestive of increased bone connectivity, improved bone trabecular structure, and a higher resistance to fracture. Similarly, a dietary 25-OHD_3_ addition exerted a positive effect on trabecular bone structure [19], tibia strength, and structure (dense cortical bone structure), as the medullary cavity was filled with numerous trabeculae [27]. Interestingly, a decline in the bone quality (bone ash, bone mineral contents, bone density, bone breaking strength, and stiffness) of broiler birds in low Ca and P level diets was reversed with the dietary 25-OHD_3_ addition [32]. Another study reported that a 56 μg/kg 25-OHD_3_ addition in the diets of pullets caused an improvement in tibia quality during the laying period [47]. Whereas the long-term dietary supplementation of 25-OHD_3_ exerted a positive effect on bone strength at 72 weeks of age but not at an earlier age [28], an indication that the duration of supplement 25-OHD_3_ in the diets of hens may play a key role in the observable effects. These results are indicative of the crucial roles of Ca and P in bone development and bone health status. It has been previously reported that a loss of trabecular bone could negatively affect bone strength [48]. Also, the capacity of 25-OHD_3_ to reduce bone deformation is due to its involvement in maintaining Ca and P homeostasis, which promotes bone mineralization. Thus, the increased bone strength might be linked to improved bone trabecular structure. Hence, a dietary 25-OHD_3_ addition can be used to improve bone fracture resistance in aged laying hens under the cage system and decrease the incidence of bone disorders.

## 5. Conclusions

In summary, dietary supplementation of 25-OHD_3_ maintained the jejunal villi morphology, increased the concentration of circulating 25-OHD_3_, sex hormones, Ca regulatory cues, and increased Ca absorption. These positive effects culminated in enhanced laying performance, egg quality, and bone health of laying hens during the late-production phase. Hence, it may be inferred that the superiority of 25-OHD_3_ over VD_3_ indicates that it has the potential to prevent age-induced osteoporosis in laying hens, thereby contributing to enhanced performance, egg quality, and blood biochemical parameters.

## Figures and Tables

**Table 1:** Composition and nutrient level of basal diet.

Ingredients	Content, %	Nutrient Level ^2^	
Corn	63.65	CP, %	16.50
Soybean meal	25.41	Metabolizable energy ^2^, MJ/kg	11.30
Soybean oil	0.11	Calcium, %	3.50
Stone powder	9.18	Total phosphorus, %	0.49
Calcium hydrophosphate	0.90	Available phosphorus, %	0.29
Salt	0.30	Met + Cys, %	0.65
DL-Methionine	0.18	Lysine, %	0.79
Premix ^1^	0.27	Threonine, %	0.56
Total	100.00	Tryptophan, %	0.18

^1^ The premix provided the following per kilogram of the diet: VA 12,500 IU, VE 15 IU, VK 2 mg, VB_1_ 1 mg, VB_2_ 8.5 mg, VB_6_ 8 mg, VB_12_ 5 mg, calcium pantothenate 50 mg, nicotinic acid 32.5 mg, biotin 2 mg, folic acid 5 mg, choline 500 mg, Mn 65 mg, I 1 mg, Fe 60 mg, Cu 8 mg, Zn 66 mg, and phytase 350 IU. ^2^ Nutrition levels were all calculated values. Metabolizable energy (ME) was calculated according to experimental diet composition and ME values of feedstuffs on poultry in the Chinese Feed Database (2018 Twenty-Ninth Edition).

**Table 2:** Effects of dietary supplementation of 25-OHD_3_ on performance of laying hens.

Items	Time/Week	25-OHD_3_ Levels, μg/kg			SEM	*p*-Value
		0	50			
		4000 IU VD_3_	2000 IU VD_3_	4000 IU VD_3_		
Average egg weight, g	1–4	62.04	61.99	61.88	0.20	0.95
	5–8	61.32	61.47	61.16	0.27	0.91
	9–12	61.14	61.91	62.12	0.55	0.60
Average daily feed intake, g	1–4	123.68	121.40	122.23	0.70	0.43
	5–8	121.24	124.34	119.44	0.98	0.11
	9–12	125.35	121.78	119.78	2.22	0.61
Feed-to-egg ratio	1–4	2.09	2.13	2.07	0.02	0.54
	5–8	2.12	2.24	2.07	0.03	0.08
	9–12	2.21	2.06	2.17	0.03	0.11
Laying rate, %	1–4	87.88	87.58	90.79	0.62	0.05
	5–8	86.63 ^b^	89.54 ^a^	87.88 ^ab^	0.19	0.01
	9–12	78.19 ^b^	84.76 ^a^	80.47 ^b^	0.83	<0.01

In the same row, values with different small letter superscripts mean significant difference (*p* < 0.05).

**Table 3:** Effects of dietary supplementation of 25-OHD_3_ on egg quality of laying hens.

Items	Time/Week	25-OHD_3_ Levels, μg/kg			SEM	*p*-Value
		0	50			
		4000 IU VD_3_	2000 IU VD_3_	4000 IU VD_3_		
Egg weight, g	4	64.43	64.39	62.87	0.55	0.54
	8	61.03	61.56	61.18	0.32	0.80
	12	62.09	62.64	62.03	0.54	0.89
Egg yolk weight, g	4	17.02	17.04	16.97	0.12	0.97
	8	16.40	16.48	16.26	0.18	0.90
	12	16.24	15.88	16.12	0.13	0.53
Egg yolk ratio,%	4	26.41	26.50	26.47	0.30	0.99
	8	26.86	26.76	26.58	0.28	0.93
	12	25.86	25.77	25.95	0.21	0.94
Albumen weight, g	4	41.11	41.13	40.11	0.49	0.65
	8	38.96	39.39	39.11	0.28	0.83
	12	40.39	40.48	39.93	0.42	0.86
Albumen ratio,%	4	63.82	63.84	63.77	0.31	1.00
	8	63.84	63.99	63.92	0.33	0.98
	12	65.04	64.63	64.35	0.23	0.51
Egg shape index	4	1.23	1.24	1.24	0.005	0.31
	8	1.31	1.32	1.32	0.005	0.99
	12	1.30	1.29	1.28	0.005	0.46
Eggshell strength, N	4	37.60	39.32	40.00	0.45	0.07
	8	33.32 ^b^	33.19 ^b^	36.66 ^a^	0.44	<0.01
	12	33.36 ^b^	35.14 ^a^	34.21 ^ab^	0.28	0.02
Thick albumen height, mm	4	6.44 ^b^	6.99 ^a^	7.09 ^a^	0.09	<0.01
	8	6.16 ^b^	7.07 ^a^	7.07 ^a^	0.12	<0.01
	12	6.42 ^b^	7.50 ^a^	7.45 ^a^	0.14	<0.01
Haugh unit	4	75.89 ^b^	81.33 ^a^	82.37 ^a^	0.73	<0.01
	8	77.02 ^b^	83.53 ^a^	83.30 ^a^	0.85	<0.01
	12	79.17 ^b^	85.32 ^a^	84.77 ^a^	0.74	<0.01

In the same row, values with different small letter superscripts mean significant difference (*p* < 0.05).

**Table 4:** Effects of dietary supplementation of 25-OHD_3_ on serum biochemical indicators of laying hens.

Items	25-OHD_3_ Levels, μg/kg			SEM	*p*-Value
	0	50			
	4000 IU VD_3_	2000 IU VD_3_	4000 IU VD_3_		
ALT, U/L	36.32 ^b^	41.51 ^a^	37.17 ^b^	0.77	<0.01
AST, U/L	74.12	75.67	73.64	0.76	0.56
ALP, U/L	331.83 ^a^	298.09 ^b^	291.82 ^b^	5.42	<0.01
ALB, g/L	24.32 ^b^	30.85 ^a^	29.51 ^a^	0.88	<0.01
UA, umol/L	252.31	241.99	252.09	3.73	0.46
CRE, umol/L	73.89	77.16	74.43	0.78	0.20
GLU, mmol/L	12.52	12.33	13.10	0.24	0.42
T-BIL, umol/L	32.69 ^a^	26.94 ^b^	30.07 ^ab^	0.76	<0.01
Ca, mmolL	2.45 ^b^	2.64 ^ab^	2.75 ^a^	0.05	0.02
P, mmol/L	2.40 ^b^	2.84 ^a^	2.73 ^a^	0.05	<0.01

Abbreviations: ALT, alanine aminotransferase; AST, aspartate aminotransferase; ALP, alkaline phosphatase; ALB, albumin; UA, uric acid; CRE, creatinine; GLU, glucose; T-BIL, total bilirubin; Ca, calcium; P, phosphorus. In the same row, values with different small letter superscripts mean significant difference (*p* < 0.05).

**Table 5:** Effects of dietary supplementation of 25-OHD_3_ on hormone levels in serum of laying hens.

Items	25-OHD_3_ Levels, μg/kg			SEM	*p*-Value
	0	50			
	4000 IU VD_3_	2000 IU VD_3_	4000 IU VD_3_		
25-OHD_3_, ng/mL	5.34 ^b^	5.81 ^a^	5.55 ^ab^	0.08	0.02
FSH, IU/L	10.09 ^c^	16.31 ^a^	13.16 ^b^	0.65	<0.01
T, nmol/L	1.14 ^b^	1.39 ^a^	1.47 ^a^	0.04	<0.01
Progesterone, nmol/L	8.01 ^b^	9.04 ^a^	8.07 ^b^	0.15	<0.01
CA, ng/mL	5.21	5.60	5.40	0.07	0.06
COR, ng/mL	26.81 ^a^	18.25 ^b^	10.20 ^c^	1.65	<0.01
DAO, ng/mL	7.79	7.51	7.26	0.10	0.10
ET, ng/mL	11.30 ^a^	11.37 ^a^	9.75 ^b^	0.22	<0.01
PTH, pg/mL	16.88 ^ab^	17.26 ^a^	15.09 ^b^	0.38	<0.01
BGP, ng/mL	1.50 ^a^	1.29 ^b^	1.20 ^b^	0.04	<0.01
CT, pg/mL	4.28 ^a^	4.04 ^b^	3.95 ^b^	0.04	<0.01

Abbreviations: FSH, follicle-stimulating hormone; T, testosterone; CA, carbonic anhydrase; COR, cortisol; DAO, diamine oxidase; ET, endotoxin; PTH, parathyroid hormone; BGP, bone gla protein; CT, calcitonin. In the same row, values with different small letter superscripts mean significant difference (*p* < 0.05).

**Table 6:** Effects of dietary supplementation of 25-OHD_3_ on antioxidant abilities in serum of laying hens.

Items	25-OHD_3_ Levels, μg/kg			SEM	*p*-Value
	0	50			
	4000 IU VD_3_	2000 IU VD_3_	4000 IU VD_3_		
GSH-Px, U/mL	519.48 ^c^	893.23 ^a^	814.94 ^b^	39.37	<0.01
CAT, U/L	5.46 ^c^	19.95 ^a^	11.86 ^b^	1.45	<0.01
SOD, U/mL	14.92 ^b^	17.81 ^a^	15.21 ^b^	0.41	0.01
MDA, nmol/mL	21.62 ^a^	8.24 ^b^	7.90 ^b^	1.57	<0.01
Hydroxyl radical scavenging ability, U/mL	238.15 ^b^	248.32 ^a^	255.02 ^a^	2.05	<0.01
Superoxide anion scavenging ability, U/mL	435.51 ^b^	527.77 ^a^	516.36 ^a^	10.42	<0.01
T-AOC, U/mL	18.99	20.49	21.57	0.42	0.07

Abbreviations: GSH-Px, glutathione peroxidase; CAT, catalase; SOD, superoxide dismutase; MDA, malondialdehyde; T-AOC, total antioxidant capacity. In the same row, values with different small letter superscripts mean significant difference (*p* < 0.05).

## Data Availability

Data are contained in this article.
